# PROTA: A Robust Tool for Protamine Prediction Using a Hybrid Approach of Machine Learning and Deep Learning

**DOI:** 10.3390/ijms251910267

**Published:** 2024-09-24

**Authors:** Jorge G. Farias, Lisandra Herrera-Belén, Luis Jimenez, Jorge F. Beltrán

**Affiliations:** 1 Department of Chemical Engineering, Faculty of Engineering and Science, Universidad de La Frontera, Ave. Francisco Salazar 01145, Temuco 4811230, Chile; jorge.farias@ufrontera.cl (J.G.F.); l.jimenez04@ufromail.cl (L.J.); 2 Departamento de Ciencias Básicas, Facultad de Ciencias, Universidad Santo Tomas, Temuco 4780000, Chile; lherrera14@santotomas.cl

**Keywords:** machine learning, deep learning, protamine, reproduction, biotechnology

## Abstract

Protamines play a critical role in DNA compaction and stabilization in sperm cells, significantly influencing male fertility and various biotechnological applications. Traditionally, identifying these proteins is a challenging and time-consuming process due to their species-specific variability and complexity. Leveraging advancements in computational biology, we present PROTA, a novel tool that combines machine learning (ML) and deep learning (DL) techniques to predict protamines with high accuracy. For the first time, we integrate Generative Adversarial Networks (GANs) with supervised learning methods to enhance the accuracy and generalizability of protamine prediction. Our methodology evaluated multiple ML models, including Light Gradient-Boosting Machine (LIGHTGBM), Multilayer Perceptron (MLP), Random Forest (RF), eXtreme Gradient Boosting (XGBOOST), k-Nearest Neighbors (KNN), Logistic Regression (LR), Naive Bayes (NB), and Radial Basis Function-Support Vector Machine (RBF-SVM). During ten-fold cross-validation on our training dataset, the MLP model with GAN-augmented data demonstrated superior performance metrics: 0.997 accuracy, 0.997 F1 score, 0.998 precision, 0.997 sensitivity, and 1.0 AUC. In the independent testing phase, this model achieved 0.999 accuracy, 0.999 F1 score, 1.0 precision, 0.999 sensitivity, and 1.0 AUC. These results establish PROTA, accessible via a user-friendly web application. We anticipate that PROTA will be a crucial resource for researchers, enabling the rapid and reliable prediction of protamines, thereby advancing our understanding of their roles in reproductive biology, biotechnology, and medicine.

## 1. Introduction

Protamines are a group of basic proteins, rich in arginine, found in the nucleus of sperm cells in many animals, including fish and mammals. Their primary function is to compact sperm DNA during spermatogenesis, which helps protect and stabilize the DNA and facilitates its transport during fertilization [1,2,3]. The study of protamines offers valuable insights into the regulation of gene expression, chromosome stability, and the mechanisms underlying male infertility [2,4,5]. Furthermore, the application of protamines extends beyond reproductive biology. Their unique properties are utilized in biotechnology and medicine, particularly in formulating drug delivery complexes and as potential therapeutic agents for various diseases [1,6,7]. Despite the importance of these proteins, their identification by conventional methods remains a challenge due to several factors. Protamines are predominantly studied in certain types of organisms, such as fish (e.g., salmon) and some mammals. However, not all organisms produce protamines, and in those that do, these proteins tend to be quite species-specific [1,8,9]. Additionally, protamines exhibit high variability and complexity between different species and even among individuals of the same species [1,9,10]. This variability complicates the annotation and confirmation of protamine sequences in omics databases.

Addressing these challenges, machine learning (ML) and deep learning (DL) offer transformative potential for enhancing protamine research. These technologies can automate the detection and classification of protein sequences, even from noisy or incomplete data, by learning from patterns identified in datasets [11,12,13]. The use of ML-based approaches to develop predictive models for proteins and peptides in the field of reproduction has been relatively unexplored in recent years. However, several models following this approach have been developed, indicating significant potential impact in this field of study [14,15,16,17].

Considering all the above, ML algorithms could be trained with known protamine sequences to predict the presence of these proteins in new omics samples with high performance. Applying machine learning and deep learning in protamine research represents a promising avenue to overcome current challenges and expand our understanding of these essential proteins. By leveraging the power of these technologies, researchers can gain deeper insights into the biological roles of protamines, their evolutionary diversity, and their potential applications in biotechnology and medicine. Consequently, this study evaluates the proposal of a protamine predictive model based on machine learning and deep learning approaches.

## 2. Results

### 2.1. Performance in Cross-Validation with Native Data

In the results of the ten-fold cross-validation on the native imbalanced training data (Table 1), the RF and KNN algorithms demonstrated the best overall performance with an ACC of 0.990. Other algorithms also showed high performance, such as MLP, XGBOOST, and LR, achieving accuracies of 0.988. The RF algorithm showed the highest recall (1.0) and F1 score (0.994), while NB achieved the highest precision (1.0). KNN demonstrated the highest kappa (0.984) and MCC (0.977) values. The RBF-SVM algorithm showed the lowest performance with an accuracy of 0.965 and a recall of 1.0.

### 2.2. Performance on the Independent Test Set with Native Data

During the testing stage using the native independent dataset (Table 2), the performance of most algorithms improved further. RF, KNN, and NB achieved perfect scores across all metrics (1.0). LIGHTGBM, MLP, XGBOOST, and LR showed nearly perfect performance with an accuracy of 0.992 and a recall of 0.999. The RBF-SVM again demonstrated the lowest performance, albeit still high, with an accuracy of 0.931 and a recall of 1.0.

While the native dataset demonstrated exceptionally high performance across various metrics, it is crucial to consider the potential impact of class imbalance on these results. Our dataset comprises 221 protamine sequences and 431 non-protamine sequences, presenting a moderate imbalance that could influence the interpretation of certain metrics. In this context, data-balancing techniques like SMOTE and GANs prove valuable by allowing for a more comprehensive evaluation of our models. These techniques help mitigate potential bias towards the majority class, enhance feature representation, and provide insights into model stability across different data distributions. Although the native dataset already yields good results, the application of SMOTE and GANs allows us to validate the robustness of our models and ensures their potential applicability in diverse scenarios.

In this study, the terms “real data” and “original data” are used interchangeably to refer to the initial, unaugmented dataset comprising 221 protamine sequences and 431 non-protamine sequences. These are the experimentally verified protein sequences collected for this research. In contrast, “synthetic data” refers to the artificially generated sequences created through SMOTE or GAN techniques to balance the dataset. When comparing real (or original) data with synthetic data, we are evaluating the differences between the biologically derived sequences and those artificially generated by our augmentation methods. This distinction is crucial for assessing the impact of data augmentation on model performance and the generalization capabilities of our protamine prediction approach.

### 2.3. Data Augmentation Using SMOTE

**Figure 1:**
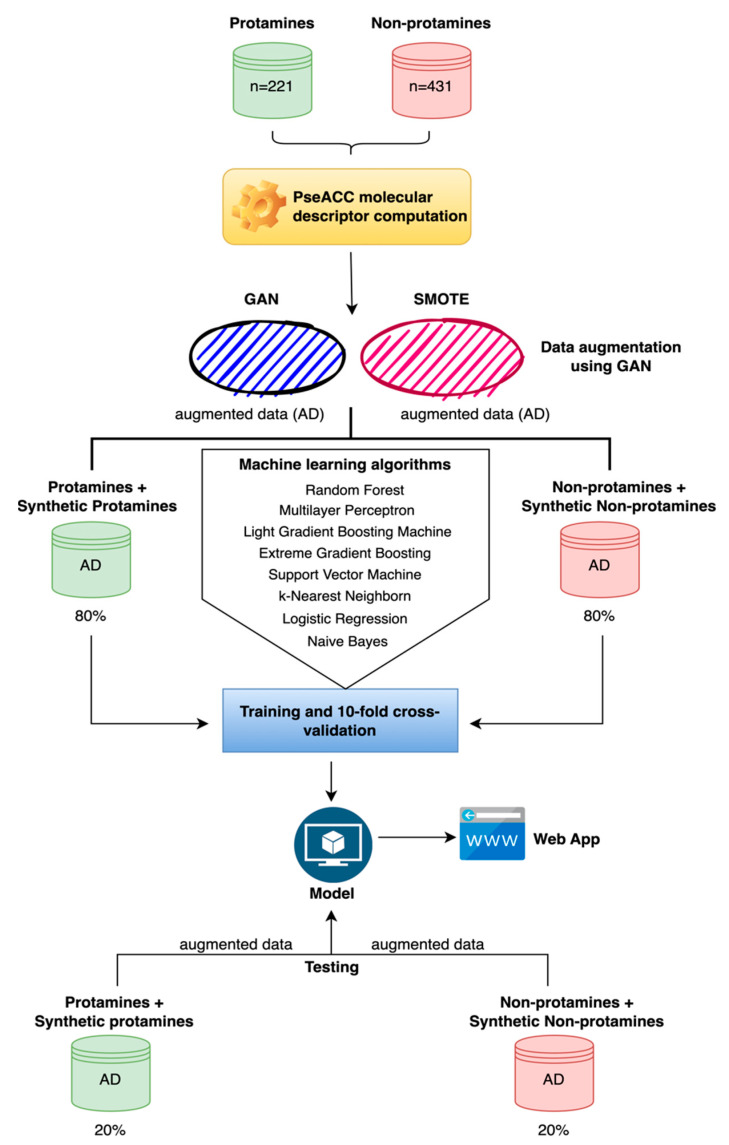
General workflow employed in this study. Sequences of real protamines and non-protamines were initially used to compute PseACC molecular descriptors. These descriptors were then utilized to generate augmented data using two different methods: Generative Adversarial Networks (GAN) and Synthetic Minority Over-sampling Technique (SMOTE). The augmented datasets were combined with the original data, forming a comprehensive dataset. This dataset was subsequently divided, with 80% allocated for training and 10-fold cross-validation of various machine learning algorithms, including Random Forest, Multilayer Perceptron, Light Gradient Boosting Machine, Extreme Gradient Boosting, Support Vector Machine, k-Nearest Neighbors, Logistic Regression, and Naive Bayes. The models were then tested on the remaining 20% of the data. Based on the evaluation of the performance metrics, the optimal model was selected and integrated into a web application.

The results of the principal component analysis (PCA) applied to both the original data and the data synthesized using the SMOTE technique are shown in Figure 1. The two-dimensional visualization of the first two principal components allows for observing the distribution of both data populations. The original data (represented in blue) show a distribution mostly clustered around the center, while the data generated by SMOTE (represented in red) are more dispersed but still maintain a pattern similar to the original data. This suggests that SMOTE is effective in generating synthetic data that reflect the general characteristics of the original data distribution, albeit with some additional variability that can be beneficial for training machine learning models.

### 2.4. Data Augmentation Using GAN

**Figure 2:**
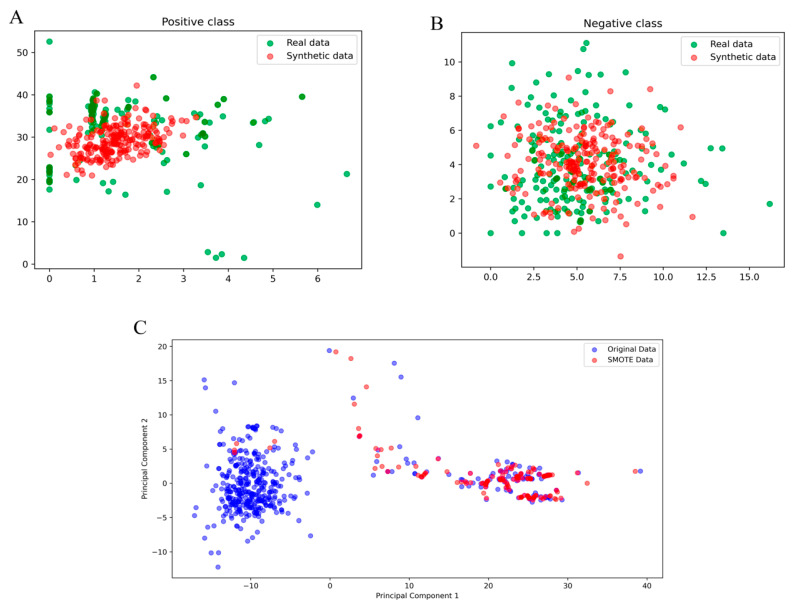
Visualization of synthetic data generated by a Generative Adversarial Network (GAN) and by Synthetic Minority Over-sampling Technique (SMOTE). Panels (**A**,**B**) show the comparison of real and synthetic data for the positive and negative classes, respectively, using a GAN. Panel (**C**) shows the comparison of original and SMOTE-generated data in a principal component space. Real data are represented in green and synthetic data in red in panels (**A**,**B**), while in panel (**C**), the original data are in blue and SMOTE-generated data are in red.

A comparison between the real data and the synthetic data for the positive class is illustrated in Figure 2. In this figure, the real data are represented in green, while the synthetic data generated by the GANs are in red. The synthetic data show significant overlap with the real data, indicating that the generation model has adequately captured the essential characteristics of the positive class. However, greater dispersion is also observed in the synthetic data, which could suggest greater diversity in the generated examples. This additional variability can be beneficial for improving the generalization of predictive models trained with this data (Figure 2).

### 2.5. Performance in Cross-Validation with SMOTE

In the results of the ten-fold cross-validation (Table 3) on imbalanced training data treated with SMOTE, the LIGHTGBM algorithm demonstrated the best overall performance with an ACC of 0.996, a recall of 0.994, a precision of 0.994, an F1 score of 0.994, a Kappa of 0.991, and an MCC of 0.991. Other algorithms also showed competitive performance, such as MLP, RF, and XGBOOST, achieving accuracies of 0.994, 0.994, and 0.992, respectively. However, the RBF-SVM algorithm showed the lowest performance with an accuracy of 0.969 and a recall of 0.909.

### 2.6. Performance on the Independent Test Set with SMOTE

During the testing stage using an independent dataset treated with SMOTE (Table 4), the LIGHTGBM, MLP, RF, XGBOOST, KNN, and LR algorithms each achieved an ACC of 0.992, a recall of 1.0, a precision of 0.977, and an F1 score of 0.988. In contrast, the NB and RBF-SVM algorithms showed slightly lower performance with accuracies of 0.984 and recalls of 0.954.

### 2.7. Performance in Cross-Validation with GAN

For the imbalanced training data treated with a GAN, the ten-fold cross-validation (Table 5) revealed that the XGBOOST algorithm achieved the highest performance with an ACC of 0.9971 in all metrics (recall, precision, F1, Kappa, and MCC). LIGHTGBM and RF also showed high performance with accuracies of 0.9942 and F1 scores of 0.9941. The RBF-SVM algorithm again showed the lowest performance with an accuracy of 0.987 and a recall of 0.9737.

### 2.8. Performance on the Independent Test Set with GAN

During the testing stage on an independent dataset treated with a GAN (Table 6), the MLP, KNN, and LR algorithms stood out with an ACC of 0.999, a recall of 0.999, a precision of 1.0, and an F1 score of 0.999. The LIGHTGBM, RF, and XGBOOST algorithms also showed high performance with accuracies of 0.9942 and F1 scores of 0.9942. On the other hand, the NB and RBF-SVM algorithms performed lower with accuracies of 0.9884 and 0.9711, respectively.

### 2.9. Overall Comparison

Overall, the results indicate that the use of SMOTE and GANs significantly improved the performance of all evaluated algorithms. The data balancing methods succeeded in equalizing the classes in the dataset, resulting in high values of accuracy, recall, and F1 score for most algorithms. Nevertheless, the MLP, KNN, and LR algorithms showed particularly outstanding performance during the independent test stage with a GAN, achieving nearly perfect performance in all metrics. These observations suggest that applying data balancing techniques such as SMOTE and GANs can considerably enhance the performance of machine learning models on imbalanced datasets, with GANs being particularly effective in this context.

### 2.10. Development of PROTA

As a result of this study, PROTA, a robust tool for protamine prediction, was developed. PROTA incorporates the best-performing predictive model obtained in our experiments, based on the MLP algorithm trained with GAN-augmented data. This tool was implemented as a web application with a minimalist and user-friendly interface, designed to be accessible to researchers without programming experience. PROTA accepts amino acid sequences as input and provides binary predictions (YES for protamines, NO for non-protamines), accompanied by a probabilistic score between zero and one, where values closer to one indicate more robust predictions. This tool represents a practical application of our findings, offering the scientific community a fast and accurate method for identifying potential protamines in genomic and proteomic datasets. PROTA is freely available at https://www.biochemintelli.com/PROTA accessed on 2 August 2024, allowing researchers to easily integrate this resource into their protein analysis workflows.

## 3. Discussion

The arginine clusters form the DNA-binding domains, allowing DNA–protamine complexes to condense and stabilize the spermatid genome. Protamines replace histones during spermatid maturation, protecting DNA from degradation [6]. Protamines are proteins of great importance for several reasons. They can protect DNA from degradation during sperm formation due to electrostatic interactions between DNA and protamine, which is positively charged. This is crucial for maintaining genetic stability in reproductive cells [18,19]. They are widely used in medicine as adjuvants in insulin formulations, extending their duration of action by forming complexes with insulin through electrostatic interactions [20]. Moreover, protamine nanoparticles have been shown to have outstanding immunomodulatory properties, making them promising components in new vaccine technologies, especially in RNA delivery systems for vaccines against infectious diseases and in cancer treatment [21,22,23]. However, due to the unique characteristics of protamines, extracting and analyzing them is more complex compared with other chromatin-associated proteins, for which there are numerous detailed protocols [24]. This complexity could be one of the main reasons why few protamines have been sequenced using mass spectrometry techniques to determine their primary sequence.

The SMOTE technique has been reported in several works as an effective method for dealing with balanced data derived from the computation of molecular descriptors from primary sequences of proteins and peptides [25,26,27,28,29,30,31]. On the other hand, the use of GANs for data augmentation is a more recent approach, which has proven to be very robust in generating high-quality synthetic data [32,33,34,35,36]. In this study, both methods enabled the development of models with high quality according to performance metrics, which were greater than 0.9 in all cases. However, the models obtained with the GAN-based approach showed an improvement, although not a significant one, in the evaluated performance metrics. These results demonstrate and align with previous studies on the robustness of applying both data augmentation techniques to tabular data derived from the computation of molecular descriptors [31,32,37,38].

The integration of GANs into PROTA, despite similar performance metrics with SMOTE, was based on several key considerations. GANs showed subtle performance improvements during cross-validation, with XGBOOST achieving marginally higher accuracy (0.997) compared with the best performance of SMOTE (0.996 with LIGHTGBM). While not statistically significant in our current dataset, this suggests potential for enhanced performance with larger, more diverse datasets. The ability of GANs to capture and generate complex, non-linear patterns aligns well with the intricate nature of protamine sequences, potentially capturing a wider range of variations compared with the interpolation approach of SMOTE [39,40,41]. Additionally, GANs offer superior scalability and adaptability as deep learning models, making PROTA more robust to potential increases in dataset size and complexity. This positions PROTA at the forefront of bioinformatics advancements, facilitating future improvements in protein sequence analysis.

ML-based methods offer several significant advantages over traditional sequence alignment methods based on the principle of homology. Firstly, ML algorithms can handle large volumes of data and detect complex patterns that are not evident with traditional alignment techniques. Traditional methods heavily rely on the similarity of known sequences, which can limit their effectiveness in identifying less conserved protein sequences or detecting new variants [42,43,44]. Consequently, ML-based methods are particularly relevant in the context of proteins such as protamines, which exhibit high variability and complexity across species. As an example of the application of PROTA, in this study, we conducted a large-scale analysis of unannotated sequences in the UniProt database for the first time [45]. The number of sequences analyzed was 3512, which are currently available without annotation in this database due to the deficiencies of traditional sequence alignment methods and the intrinsic characteristics of these proteins regarding their high variability and low homology mentioned earlier. From this total number of amino acid sequences, we identified 591 protamines using our best model incorporated in PROTA, which are available at https://github.com/jfbldevs/BioChemIntelli_datasets accessed on 2 August 2024, with binary labels of one for protamines and zero for non-protamines.

**Figure 3:**
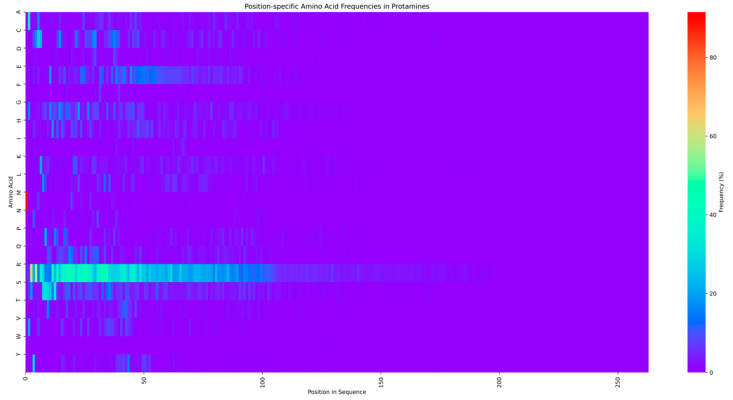
Position-specific amino acid frequencies in protamines. The heatmap displays the frequency (%) of each amino acid (Y-axis) at each position in the sequence (X-axis) of the analyzed protamines. Warmer colors indicate higher frequency, with yellow representing the highest frequencies (close to 100%) and dark purple the lowest (close to 0%). A clear predominance of arginine (R) is observed at multiple positions, especially in the N-terminal and central regions of the sequences.

The protamines identified by PROTA exhibit a marked predominance of arginine residues, with a frequency approximately three-fold higher than any other amino acid (Figure 3). This arginine-rich composition is consistent with the canonical structure of protamines and their function in DNA condensation [1,9,46,47]. The high arginine content facilitates the electrostatic neutralization of DNA phosphate groups, enabling chromatin hypercondensation in spermatozoa [48,49,50].

The consistent arginine enrichment across the identified sequences validates the specificity of PROTA in protamine detection. This characteristic amino acid profile serves as a robust molecular signature, distinguishing protamines from other nuclear proteins. The conservation of this feature across diverse taxa in the sequences identified by PROTA suggests the efficacy of the tool in capturing evolutionary variants of protamines. The pronounced arginine bias in sequences identified by PROTA corroborates the accuracy and specificity of the tool in protamine detection. This finding underscores the utility of PROTA as a computational resource for protamine identification and analysis in complex sequence datasets.

Furthermore, the identification of protamines and protamine-like proteins by PROTA has implications beyond chromatin condensation. These proteins play a crucial role in environmental toxicology and reproductive health, as semen bioaccumulates pollutants that can alter sperm quality. Pollutant-induced conformational changes in protamines can affect DNA binding and chromatin structure across various species [51]. The ability of PROTA to accurately identify both protamines and protamine-like proteins positions it as a valuable tool for investigating these environmental interactions, potentially contributing to the development of biomarkers for pollution and reproductive disorders.

While PROTA demonstrates robust performance in protamine prediction, there are several avenues for the future enhancement and expansion of its capabilities. First, although our dataset is comprehensive, continuously updating it with newly discovered protamines from diverse species could further improve the tool accuracy and broaden its applicability across different taxonomic groups. Second, while our data augmentation techniques (SMOTE and GANs) have proven effective, exploring their impact on the biological relevance of synthetic sequences presents an interesting area for future research. This could potentially lead to even more sophisticated data augmentation strategies tailored specifically for proteomic data. These considerations not only highlight the current strengths of PROTA but also underscore its potential for growth and refinement, paving the way for future advancements in protamine research and computational biology.

PROTA represents a significant step forward in protamine research, offering a powerful tool for researchers in reproductive biology, evolution, and biotechnology. By leveraging the strengths of machine learning and deep learning, we have created a robust method for protamine prediction that overcomes many of the limitations of traditional approaches. This work not only enhances our ability to identify and study these crucial proteins but also opens new avenues for research in reproductive biology and beyond.

## 4. Material and Methods

### 4.1. Dataset

In this study, sequences of protamines and non-protamines were selected and downloaded from the UniProt database [45]. This process involved a meticulous manual review of each sequence, and only those meeting the following two criteria were selected: (1) amino acid sequences with reviewed notation, and (2) support from the scientific literature demonstrating the functionality of these proteins. Through this process, 221 protamine amino acid sequences (*n* = 221) and 431 randomly selected real protein sequences (*n* = 431) were identified to form the positive and negative datasets, respectively. All the amino acid sequences used in this study are available at https://github.com/jfbldevs/BioChemIntelli_datasets accessed on 2 August 2024.

### 4.2. Calculation of Pseudo Amino Acid Composition

From all the sequences selected in this study, the molecular descriptor known as pseudo amino acid composition (PseAAC) was calculated. This molecular descriptor is widely used and popular in the development of predictive models based on primary amino acid sequences. To compute PseAAC, the propy3 library (https://propy3.readthedocs.io/ accessed on 2 August 2024), developed in Python 3 (https://www.python.org/ accessed on 2 August 2024), was used. The PseAAC consists of two main parts:

1. Twenty components representing the frequency of the 20 standard amino acids in the sequence.
2. λ additional components that capture information about the order and correlations between amino acids in the sequence, where λ is an adjustable parameter (in our study, λ = 10).

The resulting vector for each sequence has a dimension of 20 + λ.

For example, for a hypothetical X sequence “ARYRCCRSTRRNRC”:

1. Amino acid frequency components:
  [0.077, 0.385, 0.077, 0.231, 0.077, 0.077, 0.077, 0, …, 0]
  where 0.077 represents the frequency of A, 0.385 that of R, etc.
2. Sequence correlation components:
  [0.1, 0.08, …, 0.01]

These values capture information about the order and interactions between amino acids. The final PseAAC vector would be the concatenation of these two sets of values. For the binary response variable, a value of 1 was assigned to confirmed protamine sequences and 0 to non-protamine sequences. Once the calculations were completed for all the sequences under study, the values were saved in comma-separated values (csv) files for further analysis.

The independent variables in this study, derived from the PseAAC, underwent minimal preprocessing. The resulting PseAAC values, being intrinsically normalized and ranging between 0 and 1, did not require further normalization or scaling. This approach leverages a key advantage of PseAAC: it provides a machine learning-ready numerical representation of protein sequences, preserving biological information while minimizing preprocessing needs. The final PseAAC values were used directly as input for our machine learning models without further transformation.

### 4.3. Approaches for Data Augmentation

In this work, we evaluated two very different approaches to deal with the moderate number of sequences and, consequently, the number of molecular descriptors calculated from them. The first approach involved the application of the synthetic minority over-sampling technique (SMOTE), a widely used method in machine learning to address the problem of class imbalance. When a dataset is imbalanced, the model tends to be biased towards the majority class, which can lead to poor performance in predicting the minority class. SMOTE helps mitigate this problem by creating synthetic samples of the minority class [52,53]. This technique was applied to an imbalanced class file composed of previously calculated PseAAC from protamines (*n* = 221) and non-protamines (*n* = 431). For the execution of SMOTE, the imbalanced-learn library (version 0.8.0, https://imbalanced-learn.org/ accessed on 2 August 2024) was used. SMOTE was initialized with automatic sampling strategy to balance the dataset, a fixed random state for reproducibility, and 5 nearest neighbors for synthetic sample construction. The technique created synthetic samples for the minority class (protamines) until it matched the number of samples in the majority class (non-protamines), resulting in a balanced dataset of 431 samples for each class. This balanced dataset was then used for training our machine learning models, ensuring equal representation of both classes and potentially improving the performance and generalizability of our predictive models.

The second approach evaluated in this study was the use of a specific generative adversarial network (GAN) architecture for tabular data [32]. This deep learning architecture consists of two neural networks that are trained simultaneously: a generator and a discriminator. The generator network comprised three dense layers (128, 128, and 30 neurons, corresponding to the PseAAC dimensions) with LeakyReLU activation (alpha = 0.01) and dropout (rate = 0.5) between layers. The discriminator network consisted of two dense layers (128 neurons each) with LeakyReLU activation and dropout, followed by a single-neuron output layer with sigmoid activation. Both networks were optimized using Adam optimizer with a learning rate of 0.0002 and beta_1 of 0.5. The GAN was trained for 1000 epochs with a batch size of 30.

While GANs are traditionally used to generate high-dimensional data such as images or audio, for tabular data, the goal is to create data samples that mimic the distribution of an original tabular dataset. In our case, data augmentation was carried out only on the minority class dataset composed of PseAAC calculated from protamines (*n* = 221). The trained generator was used to create 210 synthetic data points, achieving a balanced dataset (*n* = 431). The synthetic data were rounded to three decimal places to match the precision of the original data. For the GAN implementation, we used TensorFlow 2 (https://www.tensorflow.org/ accessed on 2 August 2024) and Keras (https://keras.io/ accessed on 2 August 2024) libraries. The quality of the generated samples was visually assessed by comparing their distribution to the real samples using scatter plots of the first two PseAAC dimensions.

SMOTE is computationally efficient and effective for continuous features, making it suitable for our PseAAC data. However, it may not capture complex, non-linear relationships in high-dimensional data. On the other hand, GANs can identify intricate patterns in data. Although more computationally intensive, GANs offer the potential to generate high-quality synthetic samples, which is particularly beneficial given the complex nature of protein sequences. Both methods were employed to provide a comprehensive evaluation of data augmentation in protamine prediction. SMOTE offers a straightforward approach for our relatively low-dimensional PseAAC data, while GANs enable the detection of subtle, non-linear patterns that simpler methods might miss.

### 4.4. Model Training, Validation, and Testing

In both situations, considering the datasets augmented with SMOTE and GAN, the data were divided to perform training followed by a 10-fold cross-validation on 80% of the data, and then testing on the remaining 20% of the data (independent dataset). During these stages, various ML algorithms were evaluated, including Light Gradient Boosting Machine (LIGHTGBM), Multilayer Perceptron (MLP), Random Forest (RF), Extreme Gradient Boosting (XGBOOST), k-Nearest Neighbors (KNN), Logistic Regression (LR), Naive Bayes (NB), and Radial Basis Function-Support Vector Machine (RBF-SVM). To evaluate the performance of the algorithms in model generation, several metrics were used as shown below. For the entire process of training, validation, and testing, the ML libraries scikit-learn (https://scikit-learn.org/ [accessed on 2 August 2024]), XGBoost (https://xgboost.readthedocs.io/ [accessed on 2 August 2024]), and LightGBM (https://lightgbm.readthedocs.io/ [accessed on 2 August 2024]) were utilized (Figure 1).

(1) $$Accuracy\left(ACC\right)=TP+TN/\left(TP+FP+FN+TN\right)$$

(2) $$Sensitivity\left(TPR\right)=TP/\left(TP+FN\right)$$

(3) $$Precision\left(PPV\right)=TP/\left(TP+FP\right)$$

(4) $$F1\;score\left(F1\right)=2TP/\left(2TP+FP+FN\right)$$

(5) $$kappa\;(\kappa )=\frac{P_{o}-P_{e}}{1-P_{e}}$$

(6) $$Matthews\;correlation\;coefficient\;(MCC)=\frac{TP\times TN-FP\times FN}{\sqrt{\left(TP+FP\right)\left(TP+FN\right)\left(TN+FP\right)\left(TN+FN\right)}}$$

In addition to the previously discussed performance metrics, we examined the area under the curve (AUC) at each phase of the predictive model evaluation. The receiver operating characteristic (ROC) curve and the AUC provide a comparative analysis of two critical metrics: the true positive rate (TPR) and the false positive rate (FPR). In this context, the TPR is equivalent to sensitivity, as mentioned earlier, while the FPR indicates the rate at which actual negatives are incorrectly classified as positives.

(7) $$FPR=FP/\left(FP+TN\right)$$

In the final stage of this project, a web application in Python 3 (https://www.python.org/ accessed on 2 August 2024) was developed to make predictions of protamines using the best predictive model generated. This application has a minimalist interface that allows for the very easy and intuitive execution of predictions, returning the results YES and NO for protamines and non-protamines, respectively. Additionally, each result includes a probabilistic score between zero and one. Probabilistic scores closer to one indicate more robust predictions (Figure 1).

## 5. Conclusions

PROTA is a robust tool for protamine prediction that uses hybrid machine learning and deep learning approaches. By using data augmentation techniques such as SMOTE and GAN, we managed to significantly improve the performance of various ML algorithms, particularly highlighting the MLP with data augmented by GAN, achieving excellent performance metrics. The implementation of PROTA as an accessible and easy-to-use web application provides the scientific community with a valuable tool for the rapid and accurate identification of protamines in genomic and proteomic datasets. This not only facilitates progress in protamine research but also opens new possibilities in biotechnology and medicine, allowing for a better understanding and application of these essential proteins. 

## Figures and Tables

**Table 1:** Ten-fold cross-validation on the imbalanced training data.

Algorithm	ACC	Recall	Precision	F1	Kappa	MCC
*LIGHTGBM*	0.986	0.996	0.982	0.979	0.979	0.969
*MLP*	0.988	0.999	0.977	0.989	0.982	0.973
*RF*	0.990	1.0	0.976	0.994	0.984	0.977
*XGBOOST*	0.988	0.998	0.982	0.984	0.982	0.973
*KNN*	0.990	0.998	0.982	0.989	0.984	0.977
*LR*	0.988	0.999	0.982	0.982	0.982	0.974
*NB*	0.986	0.992	0.960	1.0	0.978	0.968
*RBF-SVM*	0.965	1.0	0.897	1.0	0.944	0.919

**Table 2:** Testing stage on the imbalanced independent dataset.

Algorithm	ACC	Recall	Precision	F1	Kappa	MCC
*LIGHTGBM*	0.992	0.999	0.977	1.0	0.988	0.982
*MLP*	0.992	0.999	0.977	1.0	0.988	0.982
*RF*	1.0	1.0	1.0	1.0	1.0	1.0
*XGBOOST*	0.992	0.999	0.977	1.0	0.988	0.982
*KNN*	1.0	1.0	1.0	1.0	1.0	1.0
*LR*	0.992	0.999	0.977	1.0	0.988	0.982
*NB*	1.0	1.0	1.0	1.0	1.0	1.0
*RBF-SVM*	0.931	1.0	0.795	1.0	0.886	0.837

**Table 3:** Ten-fold cross-validation on imbalanced training data treated with SMOTE.

Algorithm	ACC	Recall	Precision	F1	Kappa	MCC
*LIGHTGBM*	0.996	0.994	0.994	0.994	0.991	0.991
*MLP*	0.994	0.994	0.990	0.991	0.987	0.987
*RF*	0.994	0.983	1.0	0.991	0.987	0.987
*XGBOOST*	0.992	0.977	1.0	0.988	0.982	0.983
*KNN*	0.992	0.983	0.994	0.988	0.982	0.983
*LR*	0.992	0.994	0.985	0.989	0.983	0.984
*NB*	0.986	0.960	1.0	0.979	0.969	0.970
*RBF-SVM*	0.969	0.909	1.0	0.951	0.929	0.932

**Table 4:** Testing stage on the independent dataset using SMOTE.

Algorithm	ACC	Recall	Precision	F1	Kappa	MCC
*LIGHTGBM*	0.992	1.0	0.977	0.988	0.983	0.983
*MLP*	0.992	1.0	0.977	0.988	0.983	0.983
*RF*	0.992	1.0	0.977	0.988	0.983	0.983
*XGBOOST*	0.992	1.0	0.977	0.988	0.983	0.983
*KNN*	0.992	1.0	0.977	0.988	0.983	0.983
*LR*	0.992	1.0	0.977	0.988	0.983	0.983
*NB*	0.984	0.954	1.0	0.9767	0.965	0.966
*RBF-SVM*	0.984	0.954	1.0	0.9767	0.965	0.966

**Table 5:** Ten-fold cross-validation on imbalanced training data treated with GAN.

Algorithm	ACC	Recall	Precision	F1	Kappa	MCC
*LIGHTGBM*	0.9942	0.9913	0.9971	0.9941	0.9884	0.9886
*MLP*	0.9927	0.9913	0.9943	0.9927	0.9855	0.9857
*RF*	0.9942	0.9913	0.9971	0.9941	0.9884	0.9886
*XGBOOST*	0.9971	0.9971	0.9971	0.9971	0.9942	0.9943
*KNN*	0.9927	0.9913	0.9943	0.9927	0.9855	0.9857
*LR*	0.9913	0.9942	0.9885	0.9913	0.9826	0.9827
*NB*	0.9913	0.9855	0.9971	0.9912	0.9826	0.9829
*RBF-SVM*	0.9870	0.9737	1.0	0.9866	0.9739	0.9744

**Table 6:** Testing stage on the independent dataset using GAN.

Algorithm	ACC	Recall	Precision	F1	Kappa	MCC
*LIGHTGBM*	0.9942	0.9885	1.0	0.9942	0.9884	0.9885
*MLP*	0.999	0.999	1.0	0.999	0.999	0.999
*RF*	0.9942	0.9885	1.0	0.9942	0.9884	0.9885
*XGBOOST*	0.9942	0.9885	1.0	0.9942	0.9884	0.9885
*KNN*	0.999	0.999	1.0	0.999	0.999	0.999
*LR*	0.999	0.999	1.0	0.999	0.999	0.999
*NB*	0.9884	0.9770	1.0	0.9884	0.9769	0.9771
*RBF-SVM*	0.9711	0.9425	1.0	0.9704	0.9422	0.9438

## Data Availability

PROTA datasets (https://github.com/jfbldevs/BioChemIntelli_datasets accessed on 2 August 2024).
